# Adolescent Brain Development and Progressive Legal Responsibility in the Latin American Context

**DOI:** 10.3389/fpsyg.2020.00627

**Published:** 2020-04-24

**Authors:** Ezequiel Mercurio, Eric García-López, Luz Anyela Morales-Quintero, Nicolás E. Llamas, José Ángel Marinaro, José M. Muñoz

**Affiliations:** ^1^Center of Interdisciplinary Forensic Research, Buenos Aires National Academy of Sciences, Buenos Aires, Argentina; ^2^Instituto Nacional de Ciencias Penales, Mexico City, Mexico; ^3^Criminology Program, Faculty of Law and Social Sciences, Benemérita Autonomous University of Puebla, Puebla, Mexico; ^4^Department of Law and Political Science, National University of La Matanza, San Justo, Argentina; ^5^Department of Psychology, Universidad Europea de Valencia, Valencia, Spain

**Keywords:** neurolaw, juvenile criminal behavior, juvenile criminal law, adolescent brain, legal responsibility

## Abstract

In this article, we analyze the contributions of neuroscience to the development of the adolescent brain and shed additional light on the minimum age of criminal responsibility in the context of Latin America. In neurobiology, maturity is perceived to be complex because the brain’s temporal development process is not uniform across all its regions. This has important consequences for adolescents’ behavior; in their search for the acceptance of their peers, they are more vulnerable to pressure and more sensitive to stress than adults. Their affectivity is more unstable, and they show signs of low tolerance to frustration and important emotional reactivity, with a decrease in the capacity to self-regulate. Consequently, risky behavior presents itself more frequently during adolescence, and behaviors that transgress norms and social conventions typically peak between the ages of 17 and 19 years. However, only a small percentage of young offenders escalate their behavior to committing crimes during adulthood. In comparative law, there are considerable differences in Latin American countries’ legal dispositions regarding the minimum age of criminal responsibility; Brazil, Costa Rica, and Ecuador regard the age of criminal responsibility to be 12 years, while Argentina accepts this to be 16 years. From a legal viewpoint, however, the debate about the minimum age of criminal responsibility is connected to other circumstances that, because they are still at a developmental stage, are attributed to adolescents’ rights in their decision-making and understanding of autonomy (e.g., the minimum ages for voting, alcohol consumption, and medical consent). We argue that research on the development of the adolescent brain does not provide definitive answers about the exact age required for different juridical purposes. Nonetheless, the current state of knowledge does allow for reflection on the development and maturation of adolescents and the implications for considering them criminally responsible. It also validates demands for a system that provides adolescents with greater protection and that favors their healthy integral development. In any case, although a specific minimum age is not evident, this study is disposed not to recommend lowering the age of criminal responsibility, but rather increasing it.

## Introduction

Studies of human development often define adolescence as a complex transitional phase between childhood and adulthood. However, from a neuroscientific point of view, it is not easy to define or delimit this age group; if we take into account the fact that cognitive abilities and different brain regions do not develop uniformly or simultaneously, this is even more the case. Moreover, the complex processes of brain and cognitive development are intimately influenced by culture and environment.

This complexity has also influenced the law, as is evidenced by the variety of legislation, which considers differences in the minimum age of responsibility according to various types of activities or decisions and which could also have civil and penal consequences in adulthood.

The difficulty in defining adolescence has recently been identified by the United Nations Committe on the Rights of the Child [CRC] (2016), in General Comment No. 20. This observation centers on the temporal concept of childhood, “from 10 years until the 18th birthday” (United Nations Committe on the Rights of the Child [CRC], 2016, para. 5). In the same manner, it gives perspective to the complexity of this definition, which, among other reasons, lies in the difficulty of identifying an exact age from a biological point of view. In particular, the observation notes that “different brain functions mature at different times” (United Nations Committe on the Rights of the Child [CRC], 2016, para. 5).

Following this line of thought, we can observe a great interest in the development of the adolescent brain, which forms the focus of a variety of studies that specialize in adolescence and human rights. Such is the case with UNICEF’s recent reports on adolescence in Argentina (UNICEF, 2017) and on lowering the age of criminal responsibility in Uruguay (UNICEF, 2014). At the same time, research efforts have been strengthened by projects such as the Adolescent Brain Cognitive Development Study^1^ (Feldstein Ewing et al., 2018), which follows 10,000 children between the ages of 9 and 10 years over the course of a decade, utilizing neuroimaging studies, neuropsychological evaluations, and various non-specific health investigations.

This area of knowledge, together with the development of modern neuroimaging techniques, has begun to influence different legal systems, particularly those of the Anglosphere tradition, where neuroscientific arguments have been presented in different penal cases during the last decade (Farahany, 2015; Altimus, 2017). Specifically, knowledge of how the brain grows, matures, and develops during adolescence, and its relationship with behavior, has started to influence the law (Mercurio, 2012; Steinberg, 2013; Cohen and Casey, 2014; Jones et al., 2014). For example, the Supreme Court of the United States has used arguments based on neuroscience to inform decisions about penal cases in which adolescents have been involved; this can be seen in the cases Roper v. Simmons (2005), Graham v. Florida (2010), and Miller v. Alabama (2012).

In this article, we analyze the contributions of neuroscience to knowledge of the development of the adolescent brain and shed additional light on the minimum age of criminal responsibility in the Latin American context.

## The Brain and Adolescence

It is evident that young people and adolescents are different from adults. Research articles in the field of neuroscience have shown that it is possible to ascertain, in terms of neurobiology, the reasons for these differences. The growth and development of the brain obey the interaction between genetics and the environment (nature and nurture), modeled by the characteristics of the different evolutionary stages of human development. While in the prenatal stage genes play a key role in the formation of the different brain circuits, during the stages following birth it is experiences and interaction with the environment that influences these circuits (Pascual Urzúa, 2014).

The complex demands of the environment during development require the modification of the brain’s connections. On this subject, Churchland (2012) indicates that human beings are born with immature brains and that this is actually an evolutionary advantage as it makes it possible to obtain a greater benefit from interactions with the environment, while also allowing for adaptation to the complex physical and social world. Synaptic connections (formed through synaptogenesis) are modified during different evolutionary stages, and depending on the region of the brain, they reach their maximum expansion between 2 and 7 years of age. This is followed by a process of the elimination of connections (synaptic pruning), which is widely accepted to last until the end of adolescence in the prefrontal region. There is concrete evidence to show that synaptic pruning in the prefrontal cortex also occurs between 20 and 30 years of age (Petanjek et al., 2011). Thus, it can be said that the process of expansion occurs during childhood, whereas the process of contraction and the elimination of connections occurs during adolescence and beyond and is followed by stabilization during adulthood (Giedd et al., 1999; Gogtay et al., 2004; Pascual Urzúa, 2014). The actual hypothesis about this process is that the large neuronal expansion of the connections during childhood allows children to have a broad connection with their physical, cultural, and social environment. After this time, the most requested and strengthened connections will prevail, whereas those that are less needed will be eliminated (Pascual Urzúa, 2014).

In neurobiology, maturity is perceived to be complex because the brain’s temporal development process is not uniform across all its regions. Regions related to sensory and motor activities show a pattern unlike those related to cognitive and complex affective functions, such as the executive functions (these functions are called “the most human functions of men” by Luria) or those related to the socioemotional process (e.g., empathy). In this sense, recent studies have specifically shown that the frontal lobe finishes maturing at ~30 years of age, later than the other regions (Østby et al., 2009; Tamnes et al., 2010; Petanjek et al., 2011). This has important consequences for adolescents’ behavior.

On this point, Dahl (2004) notes the existence of a paradox in adolescents’ health; while we see increased physical growth, the strengthening of the immune system, and overall better cognitive abilities compared to childhood, morbidity and mortality increase by 200% over the same period. This is connected to the difficulties associated with adolescents having to control their behavior and manage their emotions (Kelley et al., 2004; García-López and Mercurio, 2019), which makes them more vulnerable to risky behavior (Steinberg, 2004; Gardner and Steinberg, 2005; Barbalat et al., 2009; Pfeifer et al., 2011). Clear examples of this are reckless driving, alcohol and drug consumption, and violence, while such behavior may also lead to accidents, suicide, depression, eating disorders, and risky sexual behaviors (Dahl, 2004; Eaton et al., 2008). Adolescence is therefore seen as a period of great opportunity but, at the same time, great vulnerability (García-López, 2004).

This paradox is understandable because of scientific evidence from studies about the relationship between brain development and the manifestation of risk behaviors in adolescence. This is explained in the following subsections.

### Maturity Gap

As we noted above, during adolescence, the brain and cognitive abilities do not develop at the same rate. Regions that seek reward are more active and mature earlier than the regions controlling impulses. This model is known as the “dual system” or “maturational imbalance model” (Casey et al., 2008; Steinberg, 2008). Based on analyses of more than 900 individuals between the ages of 10 and 30 years, Steinberg et al. (2009a) observed that cognitive capacity, for example, logical reasoning and memory, matures by age 16 years. Nevertheless, psychosocial maturity – self-control and future orientation, especially in the presence of peers and social contexts – does not fully mature until the person is in his/her 20s. In a large sample (*N* = 5,404), which contained individuals between the ages of 10 and 30 years in 11 countries, Icenogle et al. (2019) found that during adulthood, individuals’ sensation seeking declined, and their impulse control, future orientation, and resistance to peer influence increased. This study suggests adolescents achieve the same cognitive abilities as adults at age 16 years, but their psychosocial maturity is not developed until their 20s. These results are similar to those of previous studies (Steinberg et al., 2009a, b; Chein et al., 2011; Quinn and Harden, 2013; Shulman et al., 2014). This “maturity gap” between cognitive and psychosocial development is a window of opportunity to increase the chances of making risky decisions, leading to risky behaviors during adolescence.

### Reward-Based Behavior

The evidence about the behavior incentive process has allowed researchers to identify different brain circuitry and the important role of dopamine in these circuits. In accordance with what was explained in the subsection discussing the maturity gap during adolescence, an imbalance has been found between the reward and the regulatory circuitry.

This imbalance explains the increased reward-seeking behavior in this period, which includes monetary, novel, and social rewards, as well as the dopamine system’s sensitivity to rewards (Galvan, 2010).

Considering Galvan’s review, the dopamine system is hyperresponsive or overcommitted in its response to rewards during adolescence. This increases the tendency to seek novelty and sensations (Dahl, 2004).

Several studies have found an important availability and function of dopamine during adolescence, which can be explained by the dopamine system’s high sensitivity to reward, the search for reward, and sensation-seeking behaviors (Luna et al., 2013). In addition, this situation increases the susceptibility of adolescents to the motivational properties of substance abuse (Casey and Jones, 2010). Studies, such as the one by Casey et al. (2008), have found that risk behaviors have a neurobiological correlation with the responses to rewards. Specifically, they have found that adolescents who have sexual escapades, drink excessively, practice high-impact sports, and engage in similar activities show greater activity in the nucleus accumbens–frontal cortex, especially when they play to earn money.

Luna et al. (2013) investigated the relationship between rewards and inhibitory control using incentives based on task performance. In this study, the younger participants, children and adolescents, showed more problems with inhibiting their responses compared to adults. The adolescents took longer to complete the task, but they showed high activation in the brain regions of the reward system. This supports the idea that these behaviors lead to acquiring some reward.

In another study, Palminteri et al. (2016) compared how both adults and adolescents learn to make choices based on the information they have available. The results of their research showed that teenagers focus on rewards and find it difficult to learn to avoid punishment or consider the consequences of their actions. The volunteers had to choose symbols associated with either a reward or punishment or a symbol without a consequence. After the choice was made in each task, the participants received feedback on their performance. Adults learned faster from their experience and modified their responses. They also avoided the symbols associated with punishment and learned from the feedback to make better decisions, while teenagers had more trouble doing so.

It is important to note that the evidence about reward-based behavior is most evident in contexts of heightened arousal. For example, McKewen et al. (2019) found that adolescents who had greater behavioral arousal during a task in which they argued with their parents showed a lower regulatory ability with a lower heart rate variability rate than those who had low behavioral arousal.

### Peer Pressure and Reward Sensitivity

During adolescence, peer pressure plays a key role in behavior (Currie et al., 2004; Prinstein et al., 2011). Adolescents engage in riskier behaviors when they are with their peers than when they are alone. This is known as the “peer effect” (Gardner and Steinberg, 2005; Albert and Steinberg, 2011; Albert et al., 2013; Smith et al., 2014). The “peer effect” and reward sensitivity are interrelated and have a powerful influence on adolescent risk taking. In adolescence, a social context increases activity in reward brain regions and leads to changes in the processing of rewards, which leads to risky behaviors (Chein et al., 2011; Ciranka and van den Bos, 2019). In an experimental study, Gardner and Steinberg (2005) reported that early and late adolescents took more risks on a computerized driving task when they were with their peers, although adults showed no difference in the amount of risky driving related to the social context. Somerville et al. (2011) found that adolescents are particularly sensitive to the reward-sensitizing effects of social stimuli, but they stated that this sensitization may affect their inhibitory control. Chein et al. (2011) employed functional magnetic resonance imaging (fMRI) during a video driving task and suggested that, in the presence of peers, adolescents had increased activation in their reward brain regions and evidenced higher levels of risky driving. In a recent article, Smith et al. (2018) used probabilistic gambling and go/no-go tasks while 28 adolescents (aged 15–17 years) were in an fMRI. They found an activation of the striatum and anterior insula when adolescents made risky decisions in the presence of peers, but this presence “had minimal impact on the engagement of typical cognitive control regions.” The authors state that these results support the conclusion that when adolescents are with their peers they recruit reward-processing regions. This increases their reward sensitivity and thus leads to risky decision-making, although their capacity to engage in self-control does not diminish.

Different studies have highlighted the importance of peers and peer groups in the initiation of alcohol and drug consumption (Spear, 2000; Dishion and Tipsord, 2011; Trucco et al., 2011). In their search for the acceptance of their peers, adolescents are more vulnerable to pressure and more sensitive to stress than adults. Their affectivity is more unstable, and they show signs of a low tolerance for frustration and important emotional reactivity with a decrease in their capacity for self-regulation. These characteristics affirm that adolescents lack the same level of emotional, cognitive, or behavioral maturity as adults. Adolescents make decisions differently than mature people (Kambam and Thompson, 2009), and they overestimate short-term benefits.

Using a rodent model, Logue et al. (2014) found that juvenile mice, but not adults, increased their consumption of alcohol when their peers were present. These results suggest that during adolescence the presence of peers increases reward sensitivity, and this mechanism has been conserved among mammalian species (Trezza et al., 2011; Logue et al., 2014).

### Risky Decision-Making

During adolescence, there is an important increase in behaviors that transgress norms and social conventions, peaking between the ages of 17 and 19 years (Federal Bureau of Investigation, 2003; Loeber et al., 2011). In general, adolescents commit antisocial behaviors in peer groups, and adults do so alone (Albert et al., 2013). However, only a small percentage of young offenders escalate their behavior to committing crimes during adulthood (Loeber et al., 2011).

Why does risky behavior present itself more frequently during adolescence? At present, there is important scientific evidence showing that frontal brain regions, which are related to organization, planning, and inhibitory control, are not fully developed until the end of adolescence (the third decade of life), and these regions are the last to mature (Spear, 2000; Galvan et al., 2006; Tamnes et al., 2010; Spear, 2013; Hartley and Somerville, 2015). On the other hand, regions that are reward-sensitive and regions connected to emotions are shown to be more active (Spear, 2000; Sowell et al., 2004; Toga et al., 2006; Giedd, 2008; Hartley and Somerville, 2015) and to have greater emotional reactivity (Guyer et al., 2016). This greater activity could be related to a sensitivity to focusing on possible gains in the short term, despite the negative consequences this might bring in the future. This way, the temporal distance between the maturation of both rational and emotional systems and their fragile communication generate a period of high vulnerability to risky behavior (Steinberg et al., 2009a; Icenogle et al., 2019). Different articles (Casey et al., 2008; Steinberg, 2008; Luna and Wright, 2016) know this framework as “dual systems” or “maturational imbalance.” This model states that the difference in the development of sensation-seeking behaviors and self-control leads to a preference for behaviors that seek reward, novelty, and risk (Smith et al., 2018).

Exposure to risky behaviors (such as unprotected sexual intercourse, the consumption of toxic substances, or, most critically, the antisocial behaviors that tend to occur with greater intensity during adolescence) indicates that adolescents have less behavioral capability to prevent damage, despite the presence of more developed cognitive abilities. How is this possible? Can adolescents know the theoretical consequences of their actions but still fail to effectively inhibit them? The answer is related to the interaction among environmental factors, cerebral immaturity, and a marked decrease in activity in the prefrontal regions and their neural connections. There is also a smaller response to aversive stimuli and an increase in activity registered in regions related to the reward circuit and emotional reactivity.

Not only do structural and functional modifications in the prefrontal region improve self-control, but they also improve connections in areas related to emotions, such as the limbic system, allowing for an improvement in the interaction between cognition and emotions (Steinberg, 2008). Past studies reported the areas that regulate the processing of rewards, social information, and emotions are more sensitive and more easily aroused around middle adolescence (Giedd, 2004, 2008; Blakemore and Choudhury, 2006; Poon, 2018). This effective coordination between cortical and subcortical regions and the cognition–emotion interface encourage the modulation of activations sparked by social and affective stimuli, thus allowing deliberate reasoning. Likewise, this process is bidirectional, modulating the excessively deliberate decision-making with social and emotional information (Steinberg, 2008). As noted by Steinberg (2008), these modifications put a stop to the impulsive search for sensations and give a greater resistance to peer influence. These two factors together should decrease risk-taking; this usually occurs during adulthood.

## The Minimum Age of Legal Responsibility in Latin America

The described conditions, common of development during adolescence, have been acknowledged in the international regulatory framework through different judicial documents. For example, the Convention on the Rights of the Child (United Nations General Assembly [UNGA], 1989) indicates that “the child, by reason of his physical and mental immaturity, needs special safeguards and care” and promulgates the importance of contributing to the enforcement of children’s rights to survival and healthy development.

Furthermore, Principle 2 of the Declaration of the Rights of the Child states that:

The child shall enjoy special protection, and shall be given opportunities and facilities, by law and by other means, to enable him to develop physically, mentally, morally, spiritually and socially in a healthy and normal manner and in conditions of freedom and dignity (United Nations General Assembly [UNGA], 1959).

Similarly, Article 3 of the Convention on the Rights of the Child states that every measure taken by any public or private institution of social welfare, courthouse, government authority, or regulatory body that relates to children must consider the best interests of the child. The same criterion is used for any general commentaries made by the UN Committee on the Rights of the Child and for the advisory opinions made in the Inter-American Court of Human Rights.

In the preamble of the Convention on the Rights of the Child, it is established that children require “special care,” and in Article 19 of the American Convention on Human Rights (Organization of American States [OAS], 1969), it is indicated that they ought to receive “measures of protection.” In the case of children responsible for committing crimes, the universal system of human rights has developed the United Nations Standard Minimum Rules for the Administration of Juvenile Justice (also known as the Beijing Rules), the United Nations Guidelines for the Prevention of Juvenile Delinquency (The Riyadh Guidelines), and the United Nations Rules for the Protection of Juveniles Deprived of their Liberty. All of these highlight the need to adopt measures of specific care for minors, always taking into account their vulnerable situation as a result of their immaturity, inexperience, and mid-development status.

However, from a legal point of view, full agreement regarding the ages at which the concept of childhood is applicable has yet to be reached. For example, the Convention on the Rights of the Child establishes in Article 1 that: “For the purposes of the present Convention, a child means every human being below the age of 18 years unless under the law applicable to the child, majority is attained earlier.” In this sense, international regulations establish the importance of different treatment for children and adults, suggesting 18 years old as an appropriate age to make the distinction, but offering flexibility regarding the establishment of the age of jurisdiction according to different countries’ legislation. There is general agreement on the difference between children and adults but not on the age range that distinguishes one from the other. In paragraph 3 of Article 40 of the Convention, it is asserted that participating states must try to promote, among other things, the establishment of a minimum age, so that children under that age can be presumed not to have the capacity to disobey the penal law, but a concrete minimum age is not mentioned.

From a legal viewpoint, the debate about the minimum age of criminal responsibility is connected to other circumstances that, because they are still at a developmental stage, are attributed to adolescents’ rights in their decision-making and understanding of autonomy, such as the minimum ages for voting, buying cigarettes, consuming alcohol, medical consent, and accessing contraception. It is as if, on the one hand, adolescents’ capacity to make decisions and to take responsibility for their own actions is recognized, while, on the other hand, when convenient, this is not acknowledged.

This is evident in the application of certain public policies where, for example, the legal smoking and drinking age is established at 18 years old and the legal age for accessing contraception is 14 years. In that sense, countries like the United States set a high drinking age – 21 years old – while, as shown by a recent report by the American Academy of Pediatrics, the range for the legal smoking age is recommended to be between 18 and 21 years old^2^.

These differences can also be seen in different Latin American countries, as shown in Table 1.

**TABLE 1:** Minimum ages to exercise certain rights or to consume certain substances.

Country	Criminal age (age range)	Age of majority (and voting age)	Drinking age	Smoking age
Argentina	16–18	18 Voting at 16	18	18
Belize	Data not available	Data not available	18	18
Bolivia	14–18	18	18	18
Brazil	12–18	18 Voting at 16	18	18
Chile	14–18	18	18	18
Colombia	14–18	18	18	18
Costa Rica	12–18	18	18	18
Cuba	16–18	16	Data not available	Data not available
Dominican Republic	13–18	18	18	18
Ecuador	12–18	18 Voting at 16	18	18
El Salvador	12–18	18	18	18
Guatemala	13–18	18	18	18
Honduras	12–18	21	18	21
Mexico	12–18	18	18	18
Nicaragua	13–18	18 Voting at 16	18	18
Panama	12–18	18	18	18
Paraguay	14–18	18	18	18
Peru	14–18	18	18	18
Uruguay	13–18	18	18	18
Venezuela	14–18	18	18	Data not available

*Source: Our own elaboration based on data found in Sedletzki (2016). Regarding data about minimum ages and voting ages, current civil codes from each country have been revised. The source consulted for the drinking ages was ICAP (2012). Specific regulations from each country have been revised for smoking age.*

For example, the Argentine Civil and Commercial Code (CCyC) (Ministry of Justice and Human Rights Argentina, 2014) holds the definition of child to be those who have not yet turned 13 years old and that of adolescent to be youngsters who are between 13 and 18 years of age (CCyC, art. 25); it also includes the concept of “progressive capabilities” (CCyC, art. 117). For decisions relating to health, it states that adolescents between the ages of 13 and 16 years can decide for themselves when it comes to health treatments that are either non-invasive or that present no risk to their health or lives (CCyC, art. 26). Adolescents older than 16 years are considered to be adults for decisions that relate to the care of their bodies (CCyC, art. 26). From the age of 13 upward, even if their parents oppose, adolescents can file a lawsuit if they have judicial authorization and provided that they have legal assistance during the process (CCyC, art. 678). They can also acknowledge paternity (CCyC, art. 680).

The Argentine Civil and Commercial Code establishes an interesting distinction between adolescents when it comes to types of decision-making. As shown in different publications, the same age does not necessarily mean the same capacity to perform all the acts of civic life. Progressive capacities show that while a 14-year-old adolescent has the competence to request contraception, he/she does not have it to consent to a surgical intervention (Herrera et al., 2015). In the same way, the voting age is 16 years, while the drinking and smoking age is 18 years, and the differences in the driving age depend on whether it is for motorbikes, cars, or public transportation (16, 17, and 21 years of age, respectively).

But how is it possible to reconcile the fact that adolescents are mature enough to, for example, ask for contraceptives while being younger than 18 years, or to consent to surgery and vote at 16 years, but not to smoke until 18 years or – in the area of criminal law – to be punished if they are below the age of 16 years?

In this regard, knowledge based on neuroscience explains the fact that the decision-making process depends on the type of decision, the environment, and the context in which adolescents find themselves. In other words, it can be asserted that adolescents are mature enough to make certain decisions in determinate circumstances, but not to make others.

This debate arose in the United States as a result of two cases that reached the Supreme Court; they centered on reports made by the American Psychological Association (APA), also known as the APA (Steinberg et al., 2009a). In the first case, Hodgson v. Minnesota (1990), the discussion was over an adolescent’s right to interrupt her pregnancy without previously notifying both of her parents. The APA argued that, taking into account the scientific evidence available, adolescents between the ages of 14 and 15 years showed no differences compared to adults, either in quality or in quantity, regarding logical reasoning in the comprehension of medically informed decisions (American Psychological Association, 1990). That is to say, it maintained the criterion of adolescents’ maturity to make medically informed decisions.

In Roper v. Simmons (2005), the death penalty for adolescents was abolished, and the American Psychological Association (2005) asserted that the immaturity that leads to the lesser culpability of adolescents is grounded in three aspects:

1. a lack of development of the sense of responsibility, which makes it difficult to control impulses;
2. a high vulnerability to peer pressure;
3. adolescents’ personality not yet being completely formed, causing their personality traits to be more transitory than fixed.

In this case, the APA continued to support the criterion that asserts adolescents’ immaturity as a reason to not convict them as if they were adults.

This apparent contradiction was highlighted in the Roper case, to which the APA responded by pointing out that both cases dealt with very different issues; the first regarded adolescents’ competence to consent to medically informed treatments, whereas the second related to adolescents’ culpability in criminal law, and whether they can be convicted in the same manner as adults.

As previously mentioned, in the last few years there has been an increase in evidence of the different ages at which cognitive and psychosocial abilities develop and mature in adolescents; these abilities evolve and mature in different ways. That is to say, there is a temporal gap between the development of the cognitive abilities for information processing, the prefrontal cortex, which is mostly matured by the age of 16 years, and the development of the abilities that are required for coordination between affection and cognition – cortical and subcortical connections – the maturation of which is completed at a later time (Steinberg, 2008; Icenogle et al., 2019).

The performance of intellectual and cognitive abilities will therefore not show significant improvement beyond the age of 16 years (Steinberg et al., 2009a). Meanwhile, psychosocial maturity, which is related to impulsivity, risk perception, sensation seeking, future orientation, and resistance to peer pressure, requires an effective coordination between emotions and cognition, and this occurs from the age of 20 years onward (Steinberg, 2008; Steinberg et al., 2009a). In neurobiological terms, cognitive tasks that require adequate interaction and coordination between multiple brain regions reach their development and maturity after the age of 16 years (Steinberg, 2009; Icenogle et al., 2019).

The improvement in connectivity between cortical and subcortical areas is related to the modification of susceptibility to peer pressure, which also influences risk-taking (Steinberg, 2008). Adolescents show socioemotional network activation when in the presence of their peers; this activation brings with it a decrease in self-control regulation and a greater exposure to risky behavior. This mechanism, in which peer pressure brings a greater exposure to risk-taking, occurs in the period between the ages of 19 and 20 years (Gardner and Steinberg, 2005; Steinberg and Monahan, 2007). Therefore, behavior in adolescents will differ depending on whether they are alone or with company, or if they are emotionally activated. In early adolescence, if the socioemotional circuit is not activated – for example, when adolescents are alone or in the company of an adult – there is bound to be greater cognitive control, which allows them to avoid exposure to risky situations. However, if they are accompanied by peers, or under certain conditions such as emotional activation, the socioemotional circuit is activated, which lowers their effective regulation of cognitive control. During adolescence, these circuits of cognitive control mature in such a way that, even though high socioemotional activation conditions may still be experienced during adulthood, inclinations toward risky behavior can be modulated (Steinberg, 2008).

Following this chain of ideas, in contexts where adolescents are not emotionally activated and do have time to make a decision, meaning they are “cold thinking,” although cognitive control is still in development, it is sufficient to control impulses and promote more deliberate actions (Botdorf et al., 2017). Under these conditions, risk-taking is also like that of adults; informed medical decision-making and voting come under this context. On the other hand, in contexts where adolescents are emotionally activated, or when they are with their peers and do not have time to make a decision, meaning they are “hot thinking,” adolescents find themselves in risky situations more frequently than adults (Burnett et al., 2010; Paulsen et al., 2011). Poon (2018) found a bell-shaped development curve in hot executive functions during adolescence with a peak at the ages of 14 and 15 years. The author stated that the sensitivity to reward and the risk-taking propensity were highest during this period.

In making decisions related to health, it is not only possible to consult with different doctors, but also with other specialists or parents; generally, medical decisions are not made under strict time constraints. These are the arguments put forward by the APA in the Hodgson case; when an adolescent contemplates the option of interrupting a pregnancy, she is taking time to think about her decision. During that time, she can consult with people she trusts or with different professionals (Steinberg et al., 2009a).

Some authors extend these arguments to other judicial contexts, such as the capacity to be on trial (Grisso et al., 2003), pointing out that the abilities that a person needs to be able to be tried include an understanding of the different stages of the process, the roles of each of the actors, and the meaning of the allegations, along with the ability to reason this information. They argue that, when it comes to these abilities, differences exist between adults and adolescents younger than 15 years, but not adolescents of 16 years of age.

In “hot thinking” contexts where adolescents are under pressure from their peers, under stress, and without adult supervision, the decisions they make and their behaviors are risky and reckless (Botdorf et al., 2017). In these contexts, they are less influenced by their theoretical knowledge about potentially negative consequences and so are more willing to take risks to potentially obtain short-term rewards (Hartley and Somerville, 2015). As previously shown, when adolescents are around their peers, their behavior becomes more impulsive, and the decisions they make become riskier (Hartley and Somerville, 2015). Smith et al. (2014) examined the influence of peers on adolescent risk-taking under a gambling task and found “that the presence of peers increases risky decision-making during adolescence even when explicit information about the probability of negative outcomes is provided, and even (perhaps especially) when these negative outcomes are portrayed as highly likely.” These results suggest that when adolescents are in the presence of peers, providing adolescents with information about the likelihood of negative outcomes may not be as effective as expected.

## Further Remarks on Latin American Legislation

The difficulty in the consideration of the legal responsibility of adolescents is evident when we look at cases in different countries. For example, in the case of the United States, Farahany explains:

In a triology [*sic*] of cases [i.e., *Roper v. Simmons*, *Graham v. Florida*, and *Miller v. Alabama*], the United States Supreme Court has cited to evidence about the developing juvenile brain to find it unconstitutional under the Eighth Amendment of the United States Constitution to executive juveniles, to impose life without the possibility of parole for non-homicidal offenders, or to have a mandatory scheme of life imprisonment without the possibility of parole. Since the latest of these cases, *Miller v. Alabama*, there is considerable confusion and debate by lower courts about the meaning of that ruling and the extent to which a judge must consider neuroscience when sentencing a juvenile offender (Farahany, 2015).

Regarding the United Kingdom, Catley and Claydon (2015) state that it is “unlikely that neuroscientific advances in understanding the brains of adolescents relevant to the age of criminal responsibility would appear in English case law.” The Netherlands is another interesting case:

The measure of “Placement in an Institution for Juveniles” (“Plaatsing in Inrichting Jeugdigen,” PIJ, art 77s Criminal Code) can be imposed by the court for 3 years, and can thereafter be continued by the court to a maximum of 7 years. PIJ is intended for criminal juveniles with a developmental disorder or psychological/psychiatric problems. The aim of the PIJ-measure is reintegration into society by resocialization. In the Netherlands, juveniles of 12–18 years in principle fall under juvenile criminal law. Juveniles of 16 or 17 may be sentenced according to adult criminal law. Since the new “Adolescent Criminal Law” came into effect, Apr. 1, 2014, adolescents of 18–23 years old may be sentenced according to juvenile criminal law (de Kogel and Westgeest, 2015).

In Latin America, there are numerous human rights treaties that have been ratified by the different states and that govern this matter. With this in mind, and in consonance with Article 24 of the American Convention on Human Rights, the principle of equality must be understood to be the obligation to treat equals in the same way. It also means, however, that those not under equal conditions must not receive the same legal treatment. This is one of the reasons why children, adolescents, and adults should not be treated in the same way: as has already been explained, their cognitive abilities are not the same.

That said, rights and obligations must be implemented according to their context and the consequences that they carry with them. From this point of view, the objective of the Argentinean legislation mentioned earlier, which holds that those exercises of rights that might imply a long-term consequence for children and adolescents are the last rights to be acquired, appears appropriate. If these types of decisions were made in the context of peer pressure, or any other context of “hot thinking,” it could bring legal consequences for those in this age group. Legal limitations that demand consent from the responsible adult, or a judicial decision (if the former does not give consent), allow for the protection of adolescents’ integrity and development and force them to deliberately consider or debate their decisions. At the same time, the adolescent is treated as a “subject of rights” (and not “object of rights”): if their decision is not unreasonable or does not put them into a risky situation, they can do as they will.

This does not generate conflict as long as we are referring to the exercising of rights (such as the right to vote or to have control over one’s own body) that carry inherent responsibilities. However, when we enter the realm of legal responsibilities, there are bigger differences in the legal dispositions in comparative law: Brazil, Costa Rica, and Ecuador regard 12 as the age of criminal responsibility, whereas Argentina views this age to be 16 years.

As has been noted, adolescents’ brain development is not linear, and therefore it is not (yet) possible, from a neuroscientific perspective, to define the exact moment from which a person can act with absolute cognitive capacity (or at least a capacity appropriate to criminal responsibility). While this is true, it does not detract from the fact that recent studies have indicated that the development of the brain’s executive functions is completed after the age of 21 years. Legislative debates on increasing the age of criminal responsibility are therefore needed, so that a person between the ages of 18 and 21 years will not receive the same treatment as an older adult, and so that they will not be seen as being over the minimum age of criminal responsibility.

As such, allowing a 12-year-old child to potentially be considered as criminally responsible presents a clear contradiction to the neuroscientific advances that have been made in recent decades. At the same time, this also constitutes a violation of the principle of equality as a 12-year-old child cannot receive the same legal treatment as a 16-year-old, because they are at different stages of cognitive development.

It would be wrong, however, to consider the determination of the minimum age of criminal responsibility to be the only relation between neuroscientific advances and juvenile criminal law. The increased cognitive development, the comparative decrease in the executive functions, the greater weight of peer pressure, and the underestimation of risk must also directly influence the principle of culpability and, consequently, the criminal response that an adolescent who is considered to be criminally responsible for a crime receives.

Taking this into consideration, in Latin American comparative law, it can be observed that a wide variety of socioeducational measures are considered as appropriate criminal consequences, including admonition, fines, community service, the obligation to finish schooling, apologies to victims, damage repair, and rehabilitation, among others (e.g., Chile: art. 6 from Law 20084; Colombia: art. 177 from Law 1098; Costa Rica: art. 121 from Law 7576; Ecuador: art. 378 from Law 100; Guatemala: art. 238 from Decree 27/2003; Honduras: art. 195 from Decree 73/96).

In this sense, it is necessary to highlight Law 287 of Nicaragua. In Article 95, it is stated that a person who was between the ages of 13 and 15 years at the moment of action and who was found to be criminally responsible for committing a crime will be sentenced with the application of socioeducational measures that do not involve the deprivation of freedom, whereas those who are 12 years of age or under are exempted from any criminal responsibility. At the same time, it imposes a maximum penalty of 6 years’ imprisonment for adolescents between the ages of 16 and 18 years who are criminally convicted. This legislation is in harmony not only with the supranational legislation of human rights, but also with advances in neuroscience. Indeed, through legislation of this kind, the link between the gradual increase of criminal responsibility and the development of the adolescent brain can be demonstrated. It can therefore be cited as a very good example. On the contrary, Argentinian juvenile criminal law is considered to be incompatible with the region’s current human rights treaties^3^.

## Discussion

In recent decades (1984–2017), interest in the applications of neuroscience to law, and particularly criminal law, has increased notably (Farahany, 2015). For example, in juvenile criminal law, research on the maturation, growth, and development of the adolescent brain has had a big impact on the decisions taken by the Supreme Court of the United States (Graham v. Florida, 2010; Jackson v. Hobbs, 2012; Mercurio, 2012, 2014; Miller v. Alabama, 2012; Escobar et al., 2014).

There is much scientific evidence to show that adolescents’ inherent characteristics are based on their brains’ immaturity, the result of the interactions between the different cognitive functions – still in development – environmental demands, and the context in which these present themselves. Adolescent brains do not mature homogeneously and linearly, but instead develop according to cognitive and psychosocial abilities. This explains why adolescents might show developed abilities in certain contexts or scenarios, but not in others.

In scenarios where tasks are mainly cognitive – where there is time to make decisions, it is possible to consult an adult or evaluate the different choices and alternatives, and the level of stress is low – adolescents show competence levels similar to those of an adult (cognitive maturity) (Steinberg, 2009). More complex contexts – with high stress and emotional activation, pressure from peers, or little time to think – require coordination between affectivity and cognition (psychosocial maturity), which is still immature at the age of 16.

This temporal gap between the maturity of different abilities has generated legal debates, but it also establishes the different progressive capacities of adolescents under the law. In this sense, these different capacities establish the grounds as to why adolescents can make sanitary decisions and vote at 16 years, but cannot buy alcohol or cigarettes until later.

As Steinberg (2009) has pointed out, the cognitive maturity required for decision-making needs logical reasoning and the capacity for the comprehension and processing of relevant information. Following this line of thought, it can be seen that maturity in certain aspects of judgment develops between the ages of 11 and 16 years, arising from an improvement in abstraction, deliberation, and methods of induction. These cognitive abilities, which mature between the end of childhood and the middle of adolescence, reach a peak at the age of 16 years. That is to say, in “cold thinking” contexts, there is no significant difference in the capacity to comprehend and reason information in order to make decisions between middle adolescence and adulthood. As has been mentioned, this could lay the groundwork for the argument that the age of competence to make medical decisions should be 16 years.

However, it must be highlighted that only certain aspects of judgment mature around the age of 16 years, whereas some other cognitive–intellectual aspects are influenced by the affective sphere. In that sense, connections between the brain regions that integrate cognition and emotion are still immature during middle adolescence (16 years of age). This explains why adolescents show a less developed ability to exercise effective judgment in contexts where they find themselves influenced by emotional and social variables, despite their cognitive capacities.

Most antisocial behaviors in adolescents appear within the peer group (Piquero et al., 2003). They are mostly impulsive behaviors and are not premeditated. When adolescents are with a group of peers, unsupervised, and emotionally activated, they are more sensitive to focusing on short-term rewards and less able to think about negative consequences, which debilitates their competence to make reasonable decisions (Steinberg et al., 2009a). The influence of peer pressure is therefore more intense during adolescence than during adulthood (Gardner and Steinberg, 2005).

These characteristics, which are common signs of adolescents’ immaturity, must be (and are) taken into account for the construction of public policies; there are, for example, special regulations that stipulate the age under which the sale of cigarettes and alcohol is prohibited, the minimum age for driving, and the age at which contraceptives can be accessed (Steinberg et al., 2009a). These policies can be improved in line with new scientific evidence. It has recently been recommended that the minimum age required for smoking should be raised (Farber et al., 2015), while other measures to prohibit adolescent drivers younger than 18 years from carrying passengers, or to limit their ability to do so according to the time of day, have also been suggested^4^.

When the context allows time for adolescents to decide, consult, or obtain objective information about the risks, benefits, and alternative options, or when the influence of emotions and peers can be minimized, adolescents older than 16 years are bound to be able to make more deliberate and reasonable decisions in a similar capacity to adults (Steinberg et al., 2009a). Making decisions about health, giving medical consent to take part in an investigative project, voting, and making decisions with juridical consequences are examples of such scenarios.

Taking into account the diminished responses that adolescents have to aversive stimuli, public policies of containment should be developed to act over adolescents who experiment with risk in negative situations, given that it is less probable that they would attribute any negative results to the way that they behave (Reyna and Farley, 2006).

Differences between adolescence and adulthood are also rooted in the maturation process, and in brain, cognitive, and psychological development, while also presenting ground for new arguments that discuss a differentiated criminal treatment with less culpability for adolescents, and which take their immaturity into account.

We understand that there are some aspects that it has not been possible to explore to their fullest in this medium. One such aspect concerns the cognitive abilities required to be subjected to a full criminal trial, and how these change across different ages (Kivisto et al., 2011). From a legal point of view, and based on the progressive capacities of adolescents, from the age of 16 years onward, adolescents can make decisions about their health in the same way as an adult. Studies about adolescents’ capacity to be put on trial show that a large proportion of those who are younger than 16 years experience difficulties with specific tasks of legal reasoning (Ficke et al., 2006) and in completely comprehending their rights and how to apply them. Likewise, their capacities are influenced by stress, suggestibility, and their intellectual level (Kassin, 2008). There is strong evidence that supports the idea that youngsters who are 12 years or younger have a less developed ability to comprehend and reason juridical information when compared to adolescents who are older than 16 years or adults with no psychological alteration (Ficke et al., 2006). To this effect, research has shown that 20% of adolescents between the ages of 14 and 15 years show deficient capacities comparable to adults who have no capacity to face trial for mental health reasons (Grisso et al., 2003).

When analyzing the development and maturation of adolescents, it is also important to consider the interaction between the biological and environmental aspects; examples include the impact of different factors such as poverty, stress, and traumatic situations (Auyero and Berti, 2013). Socioeconomic status is a relevant environmental factor that affects the functioning of the adolescent brain. In a recent systematic review of studies conducted with individuals between the ages of 13 and 25 years, Buckley et al. (2019) have presented evidence that socioeconomic status influences neural activation related to the processing of emotional and social stimuli. For example, negative experiences lead to a greater degree of responses, observable through the activation of the frontal cortex, in individuals with a lower socioeconomic status. Simultaneously, this review clarified that individuals with different socioeconomic statuses can show different behavioral responses even though their corresponding patterns of neural activation are similar. In any case, the manner in which socioeconomic status affects the functioning of the adolescent brain can be influenced by other factors. In this regard, a previous study has shown “that positive maternal parenting might ameliorate the negative effects of socioeconomic disadvantage on frontal lobe development (with implications for functioning) during adolescence” (Whittle et al., 2017).

In conclusion, we argue that research on the development of the adolescent brain does not provide definitive answers about the exact age required for different juridical purposes. Nonetheless, the current state of knowledge does allow for reflection on the development and maturation of adolescents and the implications for considering them criminally responsible. It also validates demands for a system that provides adolescents with greater protection and that favors their healthy integral development. In any case, although a specific minimum age is not evident, this study is disposed not to recommend lowering the age of criminal responsibility, but rather the opposite.

The relevance of building bridges of effective communication between scientific studies of human behavior, the law, and justice systems must be emphasized; this particular case concerns the relation between neuroscience and the justice system for adolescents. It is not possible to continue along parallel pathways when the issues that demand solutions are convergent. We also consider it necessary for neuroscientific analysis to be taken into consideration by jurists, and for relevant breakthroughs in other disciplines to be included in their studies. As time passes, it is important – even essential – to increase the multidisciplinary collaborations that lead to legislative approaches based on evidence and public policies with measurable indicators (e.g., through the use of neuroimaging). In other words, an ongoing connection between neuroscientific advances and the answers to social problems that have previously been addressed through the application of the law is urgently needed.

## Author Contributions

This article is an updated, extended version of a manuscript originally published in Spanish in the journal *Boletín Mexicano de Derecho Comparado* and authored by EM, EG-L, and LM-Q (see Mercurio et al., 2018; permission was granted by the journal). NL, JÁM, and JMM contributed new perspectives and material. All the listed authors amended the article and approved the final version.

## Conflict of Interest

The authors declare that the research was conducted in the absence of any commercial or financial relationships that could be construed as a potential conflict of interest.

## Footnotes

1 For further information, refer to http://abcdstudy.org.

2 https://www.aap.org/en-us/about-the-aap/aap-press-room/aap-press-room-media-center/Pages/Tobacco-and-E-Cigarettes.aspx (accessed January 10, 2020).

3 For more on this, see Inter-American Court of Human Rights, *Mendoza et al. v. Argentina* (Preliminary Objections, Merits, and Reparations), May 14, 2013 (ser. C) No. 260, para. 295.

4 A study published in 2000 recommended restrictions on vehicle passengers if the driver is younger than 18 years. This was based on the fact that for 16- and 18-years-old drivers, the risk of fatal accidents increases when they drive with other passengers in the vehicle (Chen et al., 2000).
